# Unusual Causes of Abrupt Anuria Early Post-Renal Transplantation

**DOI:** 10.1155/2015/753159

**Published:** 2015-07-13

**Authors:** Gurudev Konana Chennabasappa, Sonika Puri, Vijay Varma, Mahesh Eswarappa

**Affiliations:** Department of Nephrology, M.S. Ramaiah Medical College, Bangalore 560054, India

## Abstract

Renal transplantation using living donors has superior outcomes in comparison to deceased donor transplantation and results in immediate allograft function in a majority of cases. Rarely may allograft be nonfunctional from the beginning, or anuria is noted after a period of good urine output. Surgical causes for anuria should be high on the differential diagnosis in immediate-to-early posttransplant period, especially in an unsensitized recipient. We present two unusual causes of early onset anuria after living related renal transplantation where early surgical reexploration salvaged renal allografts with excellent long term outcomes.

## 1. Introduction

Early graft function following a living related renal allograft transplant is expected with present surgical expertise, short warm and cold ischemia time. Sudden anuria following immediate graft function and adequate urine output raises many questions. Possibility of kinking of renal artery, vascular thrombosis, and ureteric obstruction and a possibility of accelerated/hyper acute rejection are to be considered [1]. Often there is a dilemma as when to reexplore. We are presenting two case reports wherein immediate recognition of anuria and early exploration saved the graft leading to normalization of allograft function.

## 2. Case One

**Figure 1:**
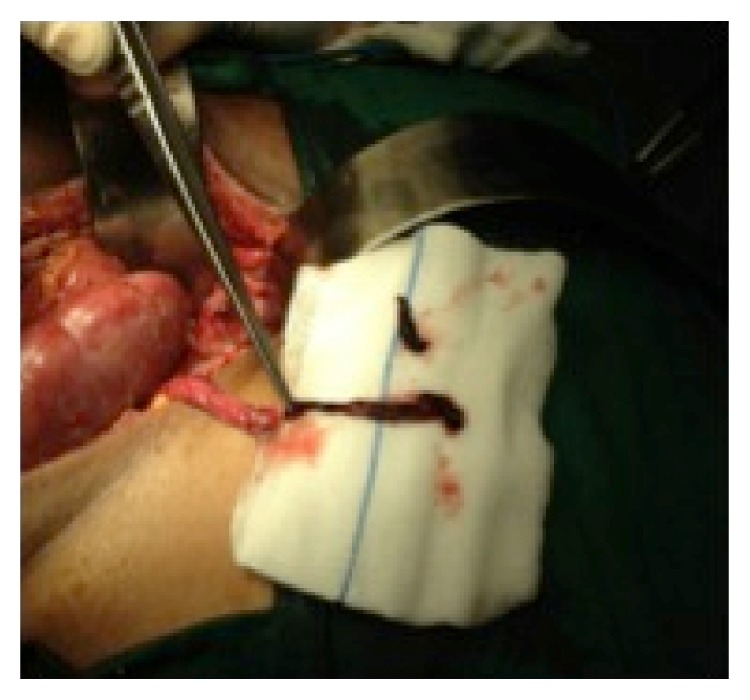
Clot retrieved from the ureter which caused the obstruction.

A thirty-six-year-old female patient, with history of end stage renal disease due to chronic glomerulonephritis, underwent a one haplomatch, living related kidney transplant from her mother. Donor kidney was retrieved by laparoscopic surgery, which was uneventful. It had single artery and vein which were anastomosed end to side to external iliac artery and vein, respectively. She received no induction except for one gram of intravenous methylprednisolone just before the release of the vascular clamps. A standard ureterovesicular anastomosis was performed with a stent in situ. Graft functioned immediately with an intraoperative urine output of 600 millilitres (mL), followed by 600 mL/hour. Mild hematuria was noted in the urinary bag. In the following half hour there was absolute anuria. A bedside Doppler ultrasound showed an empty bladder with very good perfusion to the transplanted kidney. A decision for open exploration was taken. Exploration revealed a pink and turgid transplanted kidney with a good bruit in the main renal artery and good flow in the renal vein. In contrast, the ureter appeared dusky in its entire length. Upon release of ureteric anastomosis a clot was found in the entire length of the transplant ureter (Figure 1). Brisk urine output was noted following removal of the clot. Patient's serum creatinine returned to 0.9 milligrams/decilitre (mg/dL) on postoperative day 1 with excellent urine output. Patient is currently on prednisolone, tacrolimus, and mycophenolate mofetil with excellent allograft function three years after transplant. A timely intervention saved the graft and avoided further unnecessary investigations including a renal biopsy.

## 3. Case Two

**Figure 2:**
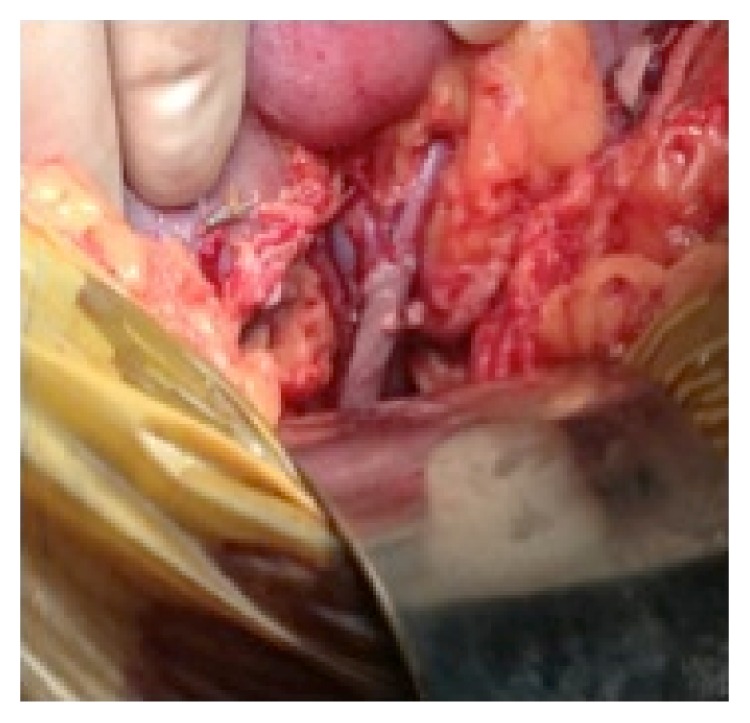
Saphenous vein graft interposed between the main renal artery and external iliac artery.

A forty-three-year-old male patient underwent a zero mismatch, living related kidney transplant in 2012. He received no induction. The donor had normal body mass index and underwent an uneventful laparoscopic donor nephrectomy. Transplant kidney had a single vein and a single artery with three hilar branches with no discernable atherosclerotic plaque. The artery and the vein were anastomosed end to side to the external iliac artery and vein, respectively. Good flow to all branches of the renal artery was noted and good urine output was noted after releasing the vascular clamps; however, sudden stoppage in urine output was noticed after closure of the surgical wound, at the time of anesthesia reversal. An urgent duplex ultrasound was performed in the operating room which revealed no flow in transplant renal artery, resulting in open exploration. Now, the allograft appeared blue and was soft on palpation. A clot was noted in the main renal artery extending in to the upper polar branch that compressed the other two branches and compromised the renal perfusion. Further, upon release of the arterial anastomosis, a dissection of the transplanted renal artery extending up to the upper pole branch was noted. The allograft was perfused with cold saline after clot extraction. The dissection was repaired in the following manner: The dissected branch of the renal artery was excised at the trifurcation of the main artery and a saphenous vein graft was interposed between the main renal artery and external iliac artery (Figure 2). At the end of the repair, upper pole of the allograft remained nonperfused. There was immediate graft function with good urine output. His serum creatinine is 1.4 mg/dL till date. Follow-up Doppler scans revealed a nonperfused upper pole with no evidence of stenosis in the saphenous vein graft. Again, a timely recognition of reduction in urine output and urgent Doppler scanning resulted in early reexploration and repair of arterial dissection, thereby averting graft loss.

## 4. Discussion

In living related renal transplantation, immediate graft function is a rule rather than an exception. On the contrary, sluggish graft function or delayed graft function is more common with deceased donor transplantation. Sudden anuria in the first few hours after surgery, especially with prior immediate graft function, raises the possibility of hyperacute rejection, kinking of the renal artery, vascular thrombosis, urinary leak, or ureteric obstruction [1, 2]. Rarely may tight closure of the abdomen lead to sudden drop in urine output [3]. Undetected calculi in the donor kidney obstructing the transplant ureter, several weeks after transplant, are known in deceased donor transplantation [4]. In most of these situations, duplex ultrasound can be diagnostic. If clinical suspicion of thrombosis is high, then a diagnostic angiogram may be necessary, although there is a risk of contrast induced nephropathy [5]. Ureteric obstruction due to organised blood clots is a rare cause of anuria and has been reported to have occurred five days after transplant in one recipient [6]. In our case, the presence of ureteric clot could be related to ureteric bleeding at the time of allograft harvesting or at the time of ureterovesicular anastomosis, although no bleeding was noted at the time of graft harvesting. Ultrasound may show either absence of hydronephrosis or presence of mild hydronephrosis, both of which may be misleading. In our first case, ureteric clot led to anuria despite the presence of a ureteric stent. Hence, sudden onset anuria in immediate-to-early posttransplant period, in a low immunologic risk recipient, with normal Doppler evaluation, should raise a suspicion of ureteric obstruction.

Vascular dissection can lead to stenosis, obstruction, and thrombosis of the graft artery and loss of graft [7, 8]. Arterial dissection associated with thrombosis could be caused due to technical reasons. Early recognition is difficult and rarely is a thrombosed graft salvaged. The causes for dissection of graft renal artery are excessive traction during harvesting and anastomosis, injury during perfusion, endothelial damage due to clamp and during anastomosis, suture techniques, and atherosclerotic arterial disease in the donor or the recipient [9]. A dissection or thrombosis of vessel should be strongly considered if Doppler ultrasonography reveals poor flow within the allograft, increased peak systolic velocity more than 200 cm/s within the main renal artery, and resistive index <0.50 indicating severe stenosis at the site of anastomosis [10]. In our case, upon discovery of arterial dissection, the decision to use saphenous vein graft was taken in order to have adequate vessel length to anastomose to a major vessel such as external iliac artery. Other options available in such situations include utilizing a segment of internal iliac artery, hypogastric artery, or, in elective cases, blood type matched cadaveric iliac artery [11, 12]. An artificial graft like Dacron graft or Polytetrafluoroethylene (PTFE) graft has also been used [13, 14]. In general, the use of saphenous vein grafts has resulted in a low but significant rate of recurrence, ranging from 0% to 12% [11].

In conclusion, surgical complications should always be considered in the differential diagnosis of abrupt onset anuria occurring several hours or days after an initial, satisfactory allograft function, especially in an unsensitized recipient. While investigations such as duplex ultrasonography and isotope renogram may eventually clinch the diagnosis, delays incurred in obtaining these investigations may diminish the chances of salvaging renal allograft especially in cases of acute vascular thrombosis. Early reexploration should be strongly considered for obtaining early diagnosis and instituting appropriate surgical management.

## References

[1] Srivastava A., Kumar J., Sharma S., Ansari M. S., Kapoor R. (2013). Vascular complication in live related renal transplant: an experience of 1945 cases. *Indian Journal of Urology*.

[2] Peter J. M., Stuart J. K. (2014). *Kidney Transplantation Principles and Practice*.

[3] Heer M. K., Trevillian P. R., Hardy D. B., Hibberd A. D. (2012). Salvaging kidneys with renal allograft compartment syndrome. *Transplant International*.

[4] Heron S. P., O'Brien D. P., Whelchel J. D., Neylan J. F. (1995). Ureteral obstruction due to calculi in the early postoperative period in renal cadaveric transplantation: a case report and discussion of ureteral obstruction in the renal transplant patient. *The Journal of Urology*.

[5] Takahashi M., Humke U., Girndt M., Kramann B., Uder M. (2003). Early posttransplantation renal allograft perfusion failure due to dissection: diagnosis and interventional treatment. *American Journal of Roentgenology*.

[6] Doehn C., Mathew A. T., Minford E. J., Kumar A., Forsythe J. L. R. (1997). Thrombotic occlusion of ureteric stent: an unusual cause of anuria after kidney transplantation. *Nephrology Dialysis Transplantation*.

[7] Tsai S.-F., Chen C.-H., Hsieh S.-R., Shu K.-H., Ho H.-C. (2013). Salvage of external iliac artery dissection immediately after renal transplant. *Experimental and Clinical Transplantation*.

[8] Khattab O. S., Al-Taee K. (2009). Early post transplantation renal allograft perfusion failure due to intimal dissection of the renal artery. *Saudi Journal of Kidney Diseases and Transplantation*.

[9] Peregrin J. H., Lácha J., Adamec M. (1999). Successful handling by stent implantation of postoperative renal graft artery stenosis and dissection. *Nephrology Dialysis Transplantation*.

[10] Chen W., Kayler L. K., Zand M. S., Muttana R., Chernyak V., DeBoccardo G. O. (2015). Transplant renal artery stenosis: clinical manifestations, diagnosis and therapy. *Clinical Kidney Journal*.

[11] Shames B. D., Odorico J. S., D'Alessandro A. M., Pirsch J. D., Sollinger H. W. (2003). Surgical repair of transplant renal artery stenosis with preserved cadaveric iliac artery grafts. *Annals of Surgery*.

[12] Rijksen J. F. W. B., Koolen M. I., Walaszewski J. E., Terpstra J. L., Vink M. (1982). Vascular complications in 400 consecutive renal allotransplants. *Journal of Cardiovascular Surgery*.

[13] Munda R., Alexander J. W., Fidler J. P., First M. R. (1976). Use of dacron vascular graft in arterial stenosis following renal allotransplantation. *American Surgeon*.

[14] Kamel M. H., Thomas A. A., Mohan P., Hickey D. P. (2007). Renal vessel reconstruction in kidney transplantation using a polytetrafluoroethylene (PTFE) vascular graft. *Nephrology Dialysis Transplantation*.

